# A decade of InsulinAPP: validation using COSMIN and clinical advancements since its initial publication

**DOI:** 10.1186/s13098-025-01717-5

**Published:** 2025-05-10

**Authors:** Marcos Tadashi Kakitani Toyoshima, Julia Mandaro Lavinas-Jones, Alina Coutinho Rodrigues Feitosa, Marcia Nery

**Affiliations:** 1 Department of Endocrinology and Metabolism, Hospital das Clínicas da Faculdade de Medicina da Universidade de São Paulo, São Paulo, Brazil; 2 Endocrine Oncology Service, Instituto do Câncer do Estado de São Paulo Octávio Frias de Oliveira, Hospital das Clínicas da Faculdade de Medicina da Universidade de São Paulo, São Paulo, Brazil; 3 Faculty of Medicine, Zarns, Salvador Brazil; 4 Endocrine Service, Hospital Santa Izabel, Santa Casa da Bahia, Salvador, Brazil

**Keywords:** InsulinAPP, Glycemic management system, COSMIN validation, Diabetes mellitus, Inpatient glycemic control, Construct validity, Digital health tools, Non-specialist physicians, Hypoglycemia prevention, Electronic glycemic management

## Abstract

This correspondence marks the 10-year milestone of InsulinAPP, a Brazilian-developed electronic glycemic management system (eGMS) designed to support inpatient insulin therapy. Initially published in 2015, InsulinAPP was developed to assist non-specialist physicians in applying evidence-based insulin protocols in hospital settings. Over the past decade, it has evolved into a validated clinical decision-support tool with demonstrated impact across multiple care contexts. In this manuscript, we present a structured overview of its validation using the COSMIN (COnsensus-based Standards for the selection of health Measurement INstruments) framework, assessing five core domains: cross-cultural adaptation, content validity, criterion validity, reliability, and construct validity. Usability testing showed high acceptance (mean Likert score 4.8/5), and expert consensus on content validity was strong (Content Validity Index = 0.95). The tool also demonstrated high reproducibility (intraclass correlation coefficient = 0.98), and in a randomized trial, glycemic control with InsulinAPP was comparable to endocrinologist-led care, with low hypoglycemia rates. Compared to other eGMS solutions, InsulinAPP stands out for its simplicity, independence from electronic health record integration, and adaptability to low-resource environments. Its protocol anticipated updates later adopted by the Endocrine Society and the Brazilian Diabetes Society, particularly regarding stratified insulinization for patients with mild-to-moderate hyperglycemia. Together, these findings confirm InsulinAPP’s scientific soundness, safety, and real-world applicability. Broader implementation and multicenter studies are warranted to further explore its impact in diverse healthcare systems and improve access to safe inpatient glycemic management.

## Introduction

Hospital hyperglycemia (HH) is a common and often underestimated challenge in inpatient care, affecting 25% of hospitalized patients, including those without a prior diagnosis of diabetes [1]. In 2015, InsulinAPP was introduced as a digital decision-support tool to assist in insulin dose calculations for hospitalized patients with diabetes mellitus (DM) or stress hyperglycemia [2]. Over the past decade, InsulinAPP has evolved into a comprehensive electronic glycemic management system (eGMS), offering support to non-specialist physicians in applying basal-bolus insulin therapy protocols.

The objective of this article is to present a structured validation of InsulinAPP using the COnsensus-based Standards for the selection of health Measurement INstruments (COSMIN) framework —a methodology increasingly applied to assess the measurement properties of digital health tools [3], including those for diabetes care [4, 5]. Additionally, we highlight the key developments and clinical advancements achieved during the first ten years of InsulinAPP’s use.

## Methods

We conducted a single-center study in Salvador, Brazil, approved by the local Ethics Committee for National Research (CAAE: 59018616.0.0000.5520). The validation of InsulinAPP followed the COSMIN framework, which recommends the evaluation of five key measurement properties: cross-cultural adaptation, content validity, criterion validity, reliability, and construct validity. Definitions and methods used for each domain are detailed below.

- Cross-Cultural Adaptation and Content Validity: These domains assess whether the application is culturally appropriate and whether its content reflects the intended clinical concepts. Structured usability testing was conducted with a diverse group of healthcare professionals (including endocrinologists, surgeons, hospitalists and internal medicine doctors) using both real and simulated scenarios. The assessment focused on six key domains: (1) Accessibility of the application, (2) Comprehension of the Portuguese language, (3) Understanding of acronyms, (4) Ease of use, (5) Objectivity, and (6) Perceived usefulness of the application. The evaluation instrument consisted of 144 multiple choice questions, divided into four sections: Initial Evaluation, Inpatient Follow-Up, Hospital Discharge, and General Evaluation of the Application. A 5-point Likert scale and the Content Validity Index (CVI) were used to assess the clarity, usability, and relevance of the tool’s content.
- Criterion Validity: This property evaluates how well InsulinAPP’s recommendations align with expert judgment. Five independent endocrinologists reviewed five hypothetical clinical scenarios and provided insulin prescriptions based on their clinical judgment and guideline adherence [6, 7]. The same cases were entered into InsulinAPP for comparison. Lin’s concordance correlation coefficient was used to assess the agreement between insulin regimens proposed by InsulinAPP and those recommended by endocrinologists. Coefficients below 0.90 were interpreted as indicating poor concordance.
- Reliability: This domain measures the consistency of results across users and time. Intra-observer reliability was assessed by three endocrinologists who independently simulated insulin management using InsulinAPP at three time points (hospital admission, 24 h, and 48 h). The endocrinologists who participated had heterogeneous professional backgrounds: one had eight years since medical graduation and one year since completion of endocrinology residency (without board certification), another had eight years since graduation and three years since residency (board-certified), and the third had 15 years since graduation and four years since residency (without board certification). This diversity reinforces the tool’s reproducibility across users with different levels of clinical experience and certification status. Two cases were intentionally duplicated to verify reproducibility.
  Inter-observer reliability was assessed using three physicians from different specialties—a hospitalist, a surgeon, and an endocrinologist—who independently used the tool to manage the same patient scenarios. Intraclass correlation coefficients (ICC) were calculated using a two-way random-effects model with absolute agreement.
  Construct Validity: This domain assesses whether the tool performs as expected in clinical practice. A randomized controlled trial (RCT) was conducted comparing 75 hospitalized patients with diabetes or stress hyperglycemia managed by non-specialist physicians using InsulinAPP versus patients managed by endocrinologists using standard protocols. Outcomes included changes in mean blood glucose from admission to discharge, hypoglycemia rates, and insulin dosing. Further methodological details and subgroup analyses related to construct validity are provided in a separate manuscript [8].

## Results

The validation of InsulinAPP using the COSMIN framework confirmed its performance across all five core measurement domains, with results summarized in Table 1:

**Table 1:** Summary of COSMIN-Based validation of InsulinAPP

COSMIN Key Domain	Objective	Key Findings
**Cross-Cultural Adaptation and Content Validity**	Assess usability, cultural alignment, and content clarity	Usability testing showed strong acceptance (Likert 4.8/5); CVI = 0.95
**Criterion Validity**	Compare InsulinAPP recommendations to expert prescriptions	Lin’s coefficient: <0.90 for insulin dosing; >0.90 for monitoring and structure
**Reliability**	Assess intra- and inter-user consistency	ICC = 0.98 (95% CI: 0.96–0.99), showing high reproducibility
**Construct Validity**	Evaluate clinical effectiveness in real-world practice	RCT showed comparable glycemic control and hypoglycemia rates (1.4%) vs. standard care

Abbreviatures: CI– confidence interval; CVI - Content Validity Index; ICC - intraclass correlation coefficient; RCT– randomized controlled trial

### Cross-cultural adaptation and content validity

Usability testing with different healthcare professionals—including endocrinologists, hospitalists, and nurses—demonstrated strong user acceptance. The average Likert score was 4.8/5, indicating excellent clarity, cultural appropriateness, and ease of use across different professional backgrounds. The CVI was 0.95, reflecting strong expert agreement with the tool’s clinical recommendations.

### Criterion validity

All Lin’s concordance correlation coefficients for insulin doses and regimens were below 0.90, whereas those related to monitoring frequency and overall treatment structure were above 0.90. These results suggest that although InsulinAPP and endocrinologist prescriptions may differ slightly in dosing, they are aligned in terms of clinical logic and recommended monitoring routines.

### Reliability

The intra-observer reliability was strong, as the insulin regimens and doses prescribed using InsulinAPP for the two identical cases were exactly the same across all evaluated time points. The ICC was 0.98 (95% CI: 0.96–0.99), confirming the tool’s reproducibility and consistent performance across different users and time points.

### Construct validity

In a randomized controlled trial involving 75 patients, mean glycemic reductions were 234 to 162 mg/dL in the InsulinAPP group and 231 to 158 mg/dL in the endocrinologist group, with hypoglycemia rates below 2%, confirming comparable outcomes [8].

## Discussion

To our knowledge, InsulinAPP is the first electronic glycemic management tool developed for inpatient use to undergo formal validation using the COSMIN framework. Originally designed to support non-specialist physicians in applying evidence-based insulin protocols, InsulinAPP demonstrated strong performance across all five COSMIN domains—cross-cultural adaptation, content validity, criterion validity, reliability, and construct validity—reinforcing its scientific robustness and real-world usability.

The COSMIN methodology, traditionally applied to health measurement instruments, proved both suitable and adaptable for evaluating a digital clinical decision-support tool [3]. By offering consistent, guideline-based insulin recommendations, InsulinAPP has the potential to overcome key barriers to glycemic control in resource-limited hospital environments, particularly where endocrinologist support is limited or absent.

Brazil has approximately 5,210 endocrinologists [9], although data on how many are actively involved in inpatient care remain scarce. This shortage is particularly concerning given the high prevalence of inpatient hyperglycemia. While most cases do not require direct specialist management, many non-specialist physicians encounter barriers such as insufficient training and lack of confidence when initiating or adjusting insulin therapy. As a result, inpatient hyperglycemia is often underdiagnosed or inadequately managed [10, 11].

In this context, InsulinAPP emerges as a scalable, validated solution that empowers non-specialists to deliver safe, evidence-based glycemic care. Importantly, the tool is not intended to replace the role of endocrinologists, but rather to extend best practices to settings where specialist input is unavailable or insufficient.

Although criterion validity showed moderate agreement between InsulinAPP and endocrinologist prescribed insulin doses, complete concordance was observed for blood glucose monitoring frequency and treatment structure. These discrepancies may be attributed to differences between the InsulinAPP algorithm and the clinical guidelines available at the time of validation [6, 7]. Since then, the 2022 Endocrine Society guideline [1] and the 2024 Brazilian Diabetes Society guideline [12] have introduced a stratified insulinization approach for patients with mild-to-moderate hyperglycemia or those on low-dose outpatient insulin therapy—an approach InsulinAPP had already incorporated from its inception [2]. This alignment underscores the tool’s foresight and ongoing relevance.

In addition to COSMIN validation, real-world studies in surgical and cardiac inpatients have demonstrated improved clinical outcomes and cost-effectiveness of InsulinAPP [13, 14]. These findings support its potential for broader implementation.

**Fig. 1:**
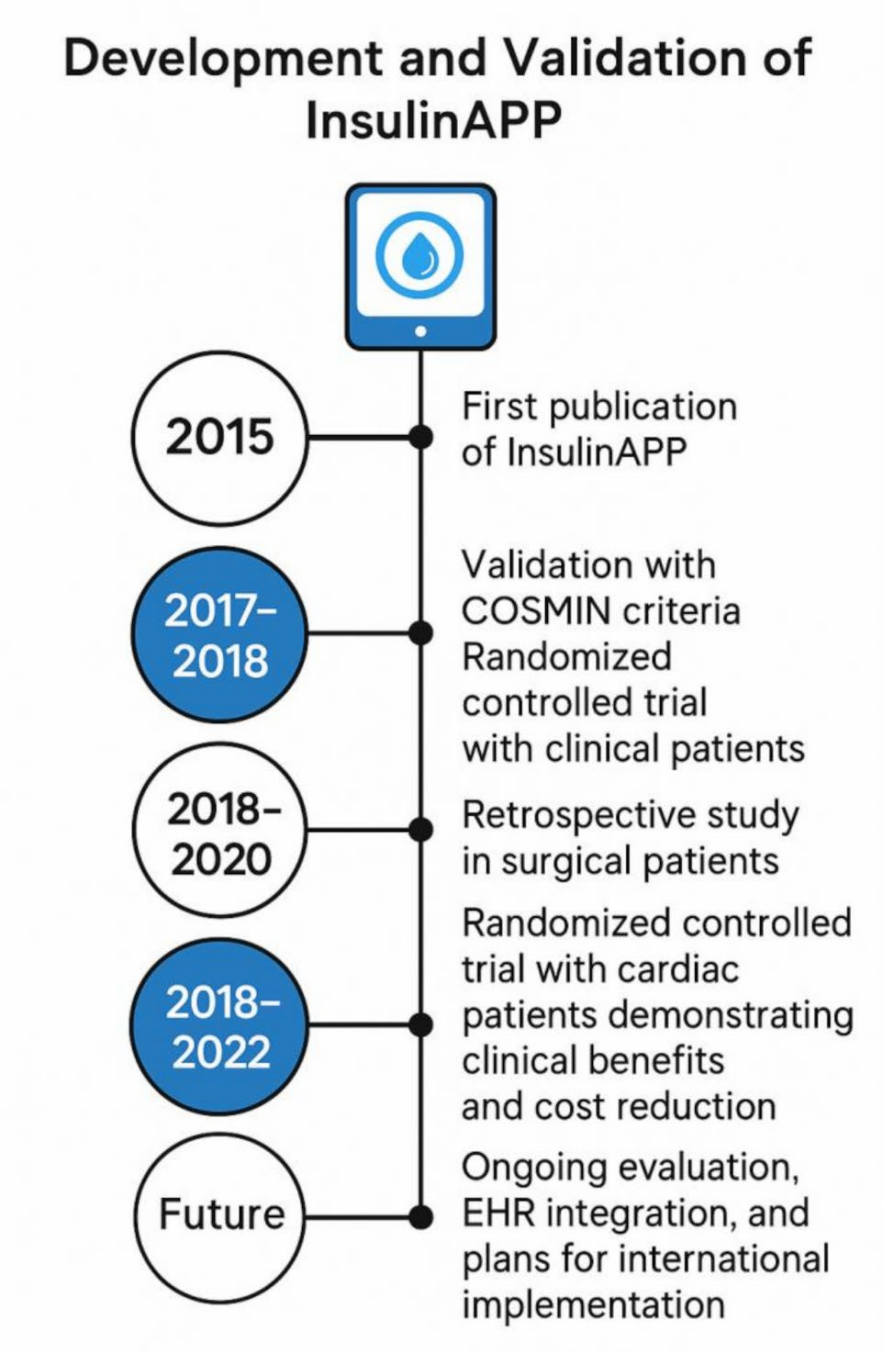
Development and validation timeline of InsulinAPP. The figure summarizes key milestones of the InsulinAPP system, from its initial publication in 2015 to ongoing plans for international implementation. The timeline includes COSMIN-based validation in clinical patients (2017–2018), a retrospective study in surgical patients (2018–2020), and a randomized controlled trial in cardiac patients (2018–2022) demonstrating clinical benefits and cost savings

The development of InsulinAPP followed a multi-phase process involving early publication [2], clinical validation, and progressive real-world implementation. Figure 1 summarizes the main milestones, including the COSMIN-based validation study in clinical inpatients, a retrospective analysis in surgical patients [13, 14], and a randomized controlled trial in cardiac patients that demonstrated both clinical efficacy and cost reduction [13].

Although the COSMIN validation was conducted in a single-center setting with predominantly clinical patients, these additional findings support the tool’s broader applicability. In particular, a previous study demonstrated the safety and effectiveness of InsulinAPP in a real-world inpatient population composed predominantly of surgical patients, further reinforcing its potential for widespread implementation in diverse hospital settings [14].

InsulinAPP has not yet been implemented outside Brazil. Future multicenter trials and international collaborations will be essential to assess generalizability across healthcare systems. Notably, its current independence from electronic health record (EHR) systems–which often requires complex infrastructures and full integration [15, 16]–makes InsulinAPP particularly well-suited for low-resource hospital settings. Future versions may include EHR integration, enhancing its applicability in hospitals with more advanced digital infrastructure.

Finally, no adverse events or unintended consequences were reported during clinical use. Hypoglycemia events were rare, and non-specialist physicians reported high confidence in using the tool after minimal training, further supporting its feasibility and safety in routine clinical practice.

## Conclusion

InsulinAPP has evolved over the past decade into a robust electronic glycemic management system, validated through the COSMIN framework across key domains of usability, validity, reliability, and clinical effectiveness. Its ability to support non-specialist physicians in delivering safe and guideline-based insulin therapy—without the need for electronic health record integration—makes it a particularly valuable tool in resource-limited hospital settings. By anticipating and aligning with recent international and national clinical guidelines, InsulinAPP demonstrates both clinical relevance and foresight. Advancing toward multicenter studies and broader implementation efforts will be essential to expand the reach of InsulinAPP and promote equitable access to high-quality inpatient diabetes care.

## Data Availability

No datasets were generated or analysed during the current study.

## Abbreviations

ADA: American Diabetes Association
CAAE: Certificate of Presentation of Ethical Appreciation
CI: Confidence interval
COSMIN: COnsensus-based Standards for the selection of health Measurement INstruments
CVI: Content Validity Index
DM: Diabetes mellitus
eGMS: Electronic glycemic management system
EHR: Electronic health record
HH: Hospital hyperglycemia
ICC: Intraclass correlation coefficient
RCT: Randomized controlled trial
